# Framing Effects on Decision-Making for Diagnostic Genetic Testing: Results from a Randomized Trial

**DOI:** 10.3390/genes12060941

**Published:** 2021-06-20

**Authors:** Andrew A. Dwyer, Hongjie Shen, Ziwei Zeng, Matt Gregas, Min Zhao

**Affiliations:** 1 William F. Connell School of Nursing, Boston College, Chestnut Hill, MA 02467, USA; 2 Massachusetts General Hospital-Harvard Center for Reproductive Medicine, Massachusetts General Hospital, Boston, MA 02114, USA; 3 Department of Measurement, Evaluation, Statistics and Assessment, Lynch School of Education, Boston College, Chestnut Hill, MA 02467, USA; edshen@foxmail.com (H.S.); ziweizeng121@gmail.com (Z.Z.); 4 Department of Research Services, Boston College, Chestnut Hill, MA 02467, USA; matt.gregas@bc.edu; 5 Carroll School of Management, Boston College, Chestnut Hill, MA 02467, USA; zhaomk@bc.edu

**Keywords:** genetic testing, genetic counselling, decision-making, choice architecture, theory of planned behavior, hereditary breast and ovarian cancer, congenital hypogonadotropic hypogonadism

## Abstract

Genetic testing is increasingly part of routine clinical care. However, testing decisions may be characterized by regret as findings also implicate blood relatives. It is not known if genetic testing decisions are affected by the way information is presented (i.e., framing effects). We employed a randomized factorial design to examine framing effects on hypothetical genetic testing scenarios (common, life-threatening disease and rare, life-altering disease). Participants (*n* = 1012) received one of six decision frames: choice, default (*n* = 2; opt-in, opt-out), or enhanced choice (*n* = 3, based on the Theory of Planned Behavior). We compared testing decision, satisfaction, regret, and decision cognitions across decision frames and between scenarios. Participants randomized to ‘choice’ were least likely to opt for genetic testing compared with default and enhanced choice frames (78% vs. 83–91%, *p* < 0.05). Neither satisfaction nor regret differed across frames. Perceived autonomy (behavioral control) predicted satisfaction (B = 0.085, *p* < 0.001) while lack of control predicted regret (B = 0.346, *p* < 0.001). Opting for genetic testing did not differ between disease scenarios (*p* = 0.23). Results suggest framing can nudge individuals towards opting for genetic testing. These findings have important implications for individual self-determination in the genomic era. Similarities between scenarios with disparate disease trajectories point to possible modular approaches for web-based decisional support.

## 1. Introduction

Technologic advances and falling costs have led to increasing use of next-generation sequencing (e.g., whole exome sequencing) in routine clinical care [1]. Genetic testing is no longer solely the domain of specialty clinics and test results are used to guide health behavior, disease management, and reproductive choices. Genetic testing decisions are challenging for patients for numerous reasons [2]. First, genetic information can be complex and difficult to comprehend without adequate genetic literacy [3]. Second, the benefit of testing may not be evident. For example, results are not always definitive (i.e., variants of unknown significance), contributing to prognostic uncertainty. Deciding to have genetic testing spurs a sequence of additional decisions arising from test results that may include preventative efforts such as significant lifestyle changes, risk-reducing surgery (e.g., hereditary breast and ovarian cancer syndrome), or reproductive decisions (e.g., pregnancy termination). Last, genetic tests are unlike other medical tests because results also implicate family members [4]. Thus, family dynamics add to the complexity of testing decisions and may contribute to decisional conflict and regret [5].

Genetic counselors have long played an important role in supporting patients and families to make informed testing decisions [6]. Broadly, the goal of genetic counseling is to support autonomy, self-determination, and high-quality decisions that are informed and aligned with individual values and preferences. The pre-test counseling process is characterized by a non-directive approach that involves providing information and focusing statements to elicit values and preferences that shape behavioral intention (decisions), while post-test counseling aims to support individuals in interpreting genetic test results. The rise of direct-to-consumer (DTC) genetic testing has altered the landscape of genetic testing as individuals make autonomous decisions outside the context of healthcare delivery [7]. Further, DTC services raise important ethical concerns because testing decisions and return of results occur without genetic counseling [8]. To date, it is unclear if genetic testing decision-making can be affected by the manner that information about testing is presented.

Choice architecture refers to the variety of ways that choices can be presented to consumers and the impact that a particular presentation has on decision-making [9]. For example, the context of a decision can be framed positively (as a gain), or negatively (as a loss) to affect decision-making in a predictable way. Choice architecture has been widely examined in consumer research and policy. Today, insights from behavior economics and choice architecture are quite well established in consumer research and policy to “nudge” people towards better financial decisions, healthier food choices, and more ecologically conscious consumption [9]. However, principles of behavioral economics and framing effects are virtually unexplored in relation to genetics. Such information would hold significant relevance for ensuring autonomy and self-determination for genetic testing decisions.

Studies of framing effects on health information messages has shown mixed results [10]. A Cochrane review found attribute framing (positive vs. negative words, e.g., “60% chance of survival” vs. “40% chance of dying”) does not influence persuasiveness yet negative goal framing (gain vs. loss, e.g., “screening will prolong your survival” vs. “not having screening will shorten your survival”) elicits more positive views of treatment effectiveness. A small proof-of-concept study compared opt-in, opt-out, and choice frames for a hypothetical oncology trial and found that the choice frame was less likely to bias preferences for participating in a hypothetical clinical trial [11]. Few studies have examined framing effects on decision-making for genetic screening. Framing had no effect on pre-conception expanded carrier screening [12]. A study examining optional bloodspot screening tests for newborns identified that the type of information provided influenced parents choosing optional testing [13]. Notably, there is a paucity of data on framing effects in the setting of diagnostic genetic testing.

We sought to determine the role of framing effects on genetic testing decision-making to inform clinical practices for pre-test genetic counseling. The Theory of Planned Behavior (TPB) [14] has been employed in the field of genetic counseling [15] to better understand and predict behaviors around prenatal genetic testing [16], testing for genetic susceptibility [17], and expanded carrier screening [12]. Guided by the TPB, we aimed to examine how presenting information in different ways (i.e., choice architecture, ‘framing’) affects cognitions/decisions of individuals facing a hypothetical genetic testing decision. The purpose of this study was threefold. First, we aimed to examine the effect of framing on genetic testing decision-making compared with a non-directive choice presentation. Second, we sought to examine the predictors of satisfaction with decision and decision regret. Last, we aimed to compare genetic testing decision-making for a common, life-threatening condition (hereditary breast and ovarian cancer, HBOC) with a rare, life-altering condition (congenital hypogonadotropic hypogonadism, CHH). Thus, we tested two null hypotheses: (i) there is no significant difference in opting for genetic testing between choice and the other frames, and (ii) there is no significant difference in opting for genetic testing between disease scenarios (HBOC vs. CHH).

## 2. Materials and Methods

This study was reviewed and approved by the Boston College Institutional Review Board (protocol 20.205.01) and the randomized trial was registered with clinicaltrials.gov (NCT04372888). This study was conducted in accordance with the Declaration of Helsinki and all participants provided opt-in electronic consent prior to study participation. Results are reported using the CONSORT-SPI 2018 extension for randomized social & psychological interventions [18].

### 2.1. Trial Design

**Figure 1:**
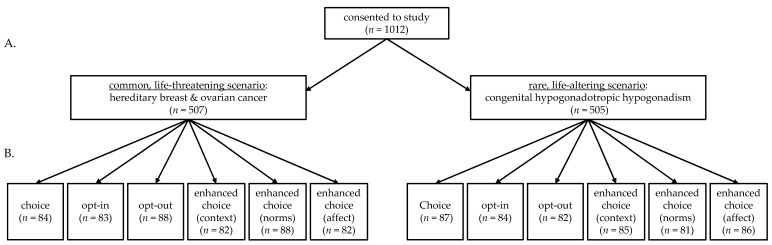
Study schematic. (**A**) Participants were randomized to one of two scenarios then reviewed information about the respective condition. (**B**) Participants were then randomized to one of six frames and made a genetic testing decision.

The study employed a randomized factorial design with two factors. The first factor was disease type (hereditary breast and ovarian cancer, HBOC, and congenital hypogonadotropic hypogonadism, CHH). The second factor was decision frame (six levels). Participants were allocated in a 1:1 ratio. After being randomized to a hypothetical genetic testing scenario (i.e., HBOC or CHH), participants were randomized to receive one of six frames for decision-making (choice, opt-in, opt-out, enhanced choice [context], enhanced choice [norms], enhanced choice [affect])—yielding 12 groups in total (Figure 1). No changes to the methods were made during the study.

### 2.2. Participants

A national sample of diverse, English-speaking adults (18+ years) living in North America were recruited (24–31 March 2020). Participants were users of Amazon’s Mechanical Turk (AMT) platform [19]. Briefly, AMT is a large, secure, web-based crowdsourcing tool for recruiting diverse participants (100,000+ members) used widely for online social and behavioral sciences research [20,21]. Studies have demonstrated AMT data and results are comparable to traditional data collection methods [22,23] and validity is supported by studies replicating the classic behavioral economics framing studies [24]. All participants provided opt-in electronic consent prior to participation in the online survey.

### 2.3. Interventions 

Following opt-in informed consent, participants provided sociodemographic information, including personal experience with breast cancer or a rare disease, and were randomized to view either the HBOC or CHH clinical scenario. Each scenario includes: (i) contextual information (i.e., hypothetical clinical information leading the individual to seek medical attention); (ii) clinical information (i.e., a summary of how the diagnosis is made, whether life threatening or life altering, treatment options, hereditary nature of the condition (that it can be passed on to offspring)), (iii) diagnostic information including approach to diagnosis (i.e., blood tests, imaging studies, with/without genetic testing, and costs) as well as possible results (i.e., making/confirming a diagnosis, effect on treatment choice, identifying at-risk blood relatives, and risk of passing on to offspring) (Supplementary Table S1).

Participants were then randomized to one of six frames and asked to make a decision about genetic testing. The wording/phrasing for each frame is provided in Supplemental Table S2. The comparator frame was active choice reflecting current genetic counseling practices. (i.e., you have two options—standard testing only or standard testing and a DNA test). Two frames were passive/default frames (opt-in, opt-out) addressing the so-called status quo bias.

The other three frames (enhanced choice) were derived from the Theory of Planned Behavior (TPB) [14]. The TPB is a well-validated framework that has been applied widely to understand and predict social and health behavior [25] that has also been applied to decision-making in genetic counseling [12,15,16,17]. The TPB posits that intention is the immediate precursor of behavior. Intention is mediated by attitudes, subjective norms, and perceived behavioral control—all of which are influenced by beliefs. Genetic tests are unlike any other test in healthcare as results implicate at-risk blood relatives. Accordingly, we hypothesized that affect/commitments, consequences, and testing norms would be important factors in decision-making. The enhanced choice frames were labelled as such because they ‘enhanced’ certain aspects of the option by making it more salient over other aspects. The three enhanced choice frames included nudges relating to affect/commitments (i.e., ability to inform at-risk blood relatives or not), testing consequences (early vs. late detection), and testing norms (what most people do) (Supplemental Table S2). Prior to launch, the survey was reviewed for health literacy and pilot tested (*n* = 6) using a qualitative “think aloud” method [26]. Briefly, individuals verbalized cognitive processes during problem-solving tasks, feedback that informed refining content presentation, and design and user engagement.

### 2.4. Outcomes

Primary outcome measures included choosing to have genetic testing (yes/no), decision satisfaction, and regret. The Satisfaction with Decision Scale (SWD) is an easy to read, validated measure of patient satisfaction with a healthcare decision across a range of conditions and patient populations [27,28]. It has good discriminant validity, correlates with decision confidence (r = 0.64), and has sufficient internal consistency (α 0.86). The decision regret scale (DRS) [29] is a brief tool (five items) that uses five-point Likert type questions to assess distress and remorse related to a healthcare decision. The DRS has good internal consistency (α 0.92). We also employed Likert type questions (7-point scale: 1 = strongly disagree and 7 = strongly agree) to assess decision cognitions (i.e., TPB motivational drivers). Questions addressed attitudes toward genetic testing (*n* = 3), subjective norms (*n* = 2) assessing norms of dyadic relationships for genetic testing (family and physician respectively), and the perceived voluntariness and ability to make a testing decision (perceived behavioral control, *n* = 3). Additionally, perceived risk (and consequences) of the condition (common vs. rare) were measured. Questions derived from the TPB had an internal consistency of (α 0.71)—generally internal consistency >0.70 is considered ‘good’. We considered that health/genetic literacy could be an important variable. As such, participants completed subjective and objective measures of health literacy. The subjective measure of health literacy [30] has been shown to detect limited health literacy as assessed by the Rapid Estimate of Adult Literacy in Medicine (REALM), a lengthier validated instrument (AUROC: 0.82) [31]. The objective measure of health literacy, Newest Vital Sign (NVS), is a brief 6-item instrument that requires individuals to identify and interpret information from a nutrition label [32]. The NVS has good internal consistency (α > 0.76) and correlates with the lengthier Test of Functional Health Literacy in Adults (TOFHLA) (AUROC: 0.88). Outcomes were measured following participant decision regarding genetic testing. No changes were made to trial outcomes after launching the study.

### 2.5. Sample Size

A power calculation was based on (common, life-threatening and rare, and life-altering) multivariate analyses testing for pairwise differences using *post hoc* t-tests adjusted for multiple comparisons using Tukey’s HSD. We assumed a significance level of 0.05. For a Cohen effect size = 0.25 (error standard deviation assumed to be 1.0). We estimated 85 subjects would be needed per treatment level combination (680 total subjects) to achieve a power level of 0.80. We set target recruitment at 1000 participants (i.e., 500 in each arm). Interim analysis was not performed and there were no stopping guidelines.

### 2.6. Randomization/Sequence Generation

Mechanical Turk users interested in participating were linked to a Qualtrics™ survey to review the informed consent. After providing consent, the Qualtrics™ program randomized participants in blocks of 12. Participants were blinded to randomization and data were not reviewed by investigators until data collection was completed.

### 2.7. Statistical Methods

We used ANOVA to assess the relationship between frames and satisfaction (SWD) and regret (DRS), respectively. One-way ANOVA was applied to detect relationships between TPB responses and frames. Scheffe and Games–Howell *post hoc* tests were used as appropriate for between-group comparisons. Student’s *t*-tests were employed to assess relationships between subjective and objective health literacy (NVS), respectively, and testing decision. Linear regression was used to assess relationships between health literacy and education (collapsed into less than college education vs. college education or more). Logistic regression was used to examine if personal family experience with breast cancer or a rare disease affected genetic testing decision. Similarly, logistic regression was used to compare genetic test decision between and across groups. Multiple linear regression was utilized to explore the relationship between TPB responses and satisfaction (SWD) and regret (DRS), respectively. Significant standardized coefficients were compared to identify the largest effect satisfaction and regret, respectively. A *p* value of < 0.05 was considered statistically significant.

## 3. Results

### 3.1. Participant Characteristics

In total, 1012 participants were recruited using Amazon’s Mechanical Turk platform (see Methods) and completed the study. Briefly, participants were randomized to one of two scenarios: either a common, life-threatening genetic condition (HBOC, *n* = 507) or a rare, life altering genetic condition (CHH, *n* = 505). After reviewing structured information (see Methods), participants were randomized to one of six frames for decision-making (Supplemental Table S2) and then declared their decision regarding genetic testing. Participants reported no harms. Participants were evenly distributed between the groups and did not differ in terms of sociodemographics (Table 1). Overall, the mean age was 36 ± 11 years (95% CI: 34.4–42.1 years), the majority of participants were male (604/1012, 60%), and 424/1012 (42%) self-identified as white. Participants were generally well educated with 690/1012 (42%) having attained a bachelor’s degree or higher and 822/1012 (81%) had adequate health literacy, subjectively. In terms of objective health literacy, the participant mean score (2.97 ± 0.06, 95% CI: 2.85–3.08) is at the high end of the intermediate range (NVS score 0–1: high likelihood of limited health literacy, 2–3: possibility of limited health literacy, 4–6: almost always indicates adequate health literacy) (32). More than half were married (536/1012, 53%) and 502/1012 (81%) reported having children.

### 3.2. Effect of Choice Architecture (Framing) on Genetic Testing Decisions

**Figure 2:**
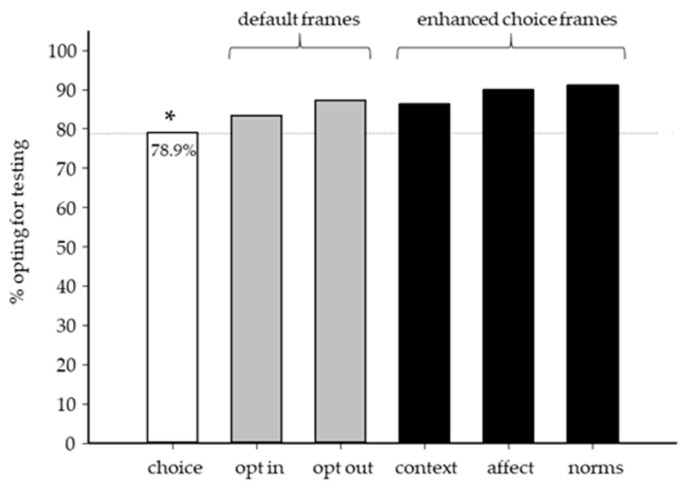
Framing effects on hypothetical genetic testing decision-making (*n* = 1012). Participants randomized to the choice frame (white) were significantly less likely to opt for testing (135/171 [78.9%], *p* < 0.05) compared with default (opt-in: 139/167 [83.2%], opt out: 148/170 [87.1%]), or enhanced choice frames (context/consequences: 144/167 [86.2%], affect/commitment: 151/169 [89.9%], norms: 154/169 [91.1%]). The gray dotted line depicts ‘choice’ as a reference point for default (gray bars) and enhanced choice frames (black bars). * *p* < 0.05.

Examining choice architecture in the overall group, participants randomized to the ‘choice’ frame (*n* = 171) were least likely to opt for testing compared with all other frames (79% vs. 83–91%, *p* < 0.05) (Figure 2). Using choice as the base, we used logistic regression to determine the magnitude of the framing effect. One passive/default frame and two enhanced choice frames exhibited significant effects. Using the ‘opt out’ frame increased the odds for choosing genetic testing (OR 1.79, *p* = 0.048). The enhanced choice frames, derived from the TPB, increased the likelihood of choosing to have genetic testing (‘norms’: OR 2.73, *p* = 0.002, ‘affect/commitment’: OR 2.36, *p* = 0.007). We hypothesized that objective health literacy (NVS) could play a role in genetic testing decisions. However, we did not observe any association between NVS and overall decision to opt for genetic testing (OR = 1.048, t = −0.462, *p* = 0.64) regardless of frame. Among frames, there was no association observed between objective health literacy and decision to opt for genetic testing. In terms of disease scenario, framing neither had an effect on satisfaction (HBOC: F = 1.819, *p* = 0.10; CHH: F = 0.699, *p* = 0.62) nor regret (HBOC: F = 1.735, *p* = 0.12; CHH: F = 1.118, *p* = 0.35). We considered that framing could affect decision cognitions—yet no significant differences were observed across frames. Specifically, decision cognitions relating to “norms” (*n* = 3) did not differ between active choice, opt-out, and enhanced choice (*p* = 0.86, *p* = 0.12, *p* = 0.29 respectively). Similarly, decision cognitions relating to “consequences” (*n* = 3) did not differ between the enhanced choice (consequences) frame and active choice (*p* = 0.66, *p* = 0.42, *p* = 0.87, respectively).

**Figure 3:**
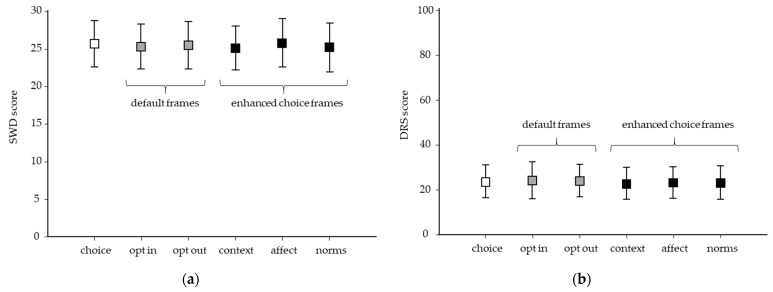
Satisfaction with decision and decision regret according to framing (*n* = 1012). Satisfaction with decision (SWD) and decision regret (DRS). (**a**) SWD scores did not differ across frames (F = 1.353, *p* = 0.24). (**b**) DRS scores did not differ across decision frames (F = 0.875, *p* = 0.49). Boxes show mean scores ± one standard deviation (error bars). White = choice, gray = default frames (opt-in, opt-out), and black = enhanced choice (context/consequence, affect/commitments, norms).

Neither satisfaction nor regret differed across the six frames (F = 1.353, *p* = 0.24; F = 0.875, *p* = 0.49, respectively) (Figure 3). As satisfaction and regret are important outcomes for genetic testing decision-making, we used multiple linear regression to identify if elements of the TPB predict satisfaction and regret, respectively (Table 2). First, we examined for collinearity to determine if multiple significant effects could be masked in the multiple linear regression. No collinearity effects were observed, therefore, we did not consider this problematic for the analysis. The TPB concept of behavioral control (i.e., “Having genetic testing is entirely up to me”) was a predictor of satisfaction (B = 0.085, *p* < 0.001). Conversely, feeling one lacked behavioral control (i.e., “I feel I have no control over my decision to have genetic testing”, B = 0.346, *p* < 0.001) predicted decisional regret. Considering genetic testing as being beneficial for family members predicted satisfaction with decision yet TPB elements relating to consequences (physical, psychological, and social) had little effect on satisfaction. Several TPB factors predicted decision regret including perceiving that the condition would affect one personally, have physical consequences, or social (e.g., discrimination) consequences. Interestingly, perceiving the decision as being “easy” was associated with both satisfaction and regret.

### 3.3. Common, Life-Altering Scenario vs. Rare, Life-Altering Scenario

Participants were randomized to make a genetic testing decision for genetic conditions with disparate consequences—either a common/life-threatening condition (hereditary breast and ovarian cancer, HBOC) or a rare, life-altering condition (congenital hypogonadotropic hypogonadism, CHH). A secondary aim of the study was to examine if differences were noted in genetic testing decisions between conditions with divergent frequencies that are at opposing ends of the lethality spectrum. In this hypothetical setting, the decision to opt for genetic testing did not differ between HBOC and CHH (443/507 [87.4%] vs. 428/505 [84.8%], respectively, *p* = 0.23). Framing neither had an effect on satisfaction (HBOC: F = 1.819, *p* = 0.10; CHH: F = 0.699, *p* = 0.62) nor regret (HBOC: F = 1.735, *p* = 0.12; CHH: F = 1.118, *p* = 0.35). We hypothesized that having experience (personal or family) with either breast cancer or a rare disease could affect testing decisions. Groups did not differ in terms of the rates of having prior experience (HBOC: 93/507 (18%), CHH: 87/505 (17%), Table 1) and rates of individuals with prior experience did not differ across frames. Logistic regression revealed that having prior experience did not have any effect on opting to have genetic testing (HBOC: OR = 0.86, 95%CI: 0.60–1.24, *p* = 0.42; CHH: OR = 0.60, 95%CI: 0.39–0.92, *p* = 0.21). Similarly, no significant differences were observed according to individual frame. Thus, individuals with prior experience of the conditions (and presumably strong views about genetic testing) do not appear to be more influenced by the frames. We compared decision cognitions (based on the TPB) between the common and rare scenarios. Within-group scores did not differ across frames and scores were similar in 11/13 decision cognition questions (Supplemental Table S3). The HBOC group assigned higher ratings than the CHH group for perceived risk (i.e., “The health scenario would affect me personally”, 5.97 ± 1.18 (95%CI: 5.86–6.07) vs. 5.77 ± 1.31 (95%CI: 5.65–5.88), *p* = 0.012) and norms (i.e., “Having genetic testing would be important for people I care about”, 5.92 ± 1.24 (95%CI: 5.81–6.03) vs. 5.72 ± 1.32 (95%CI: 5.61–5.84), *p* = 0.012). While the differences reached statistical significance, it is not clear that the magnitude of difference on a seven-point Likert-type scale would be clinically meaningful.

## 4. Discussion

Herein we present findings of an experiment examining genetic testing decision-making in two hypothetical scenarios (common/life-threatening and rare/life-altering). Traditionally, genetic counseling employs a non-directive approach (i.e., choice) to support patients and families in making testing decisions that are informed and aligned with values and preferences [6]. We observed that default frames (i.e., opt-in, opt-out) as well as enhanced choice frames (based on the TPB) all increased the likelihood of individuals opting for genetic testing compared with the ‘choice’ frame. Findings from these hypothetical testing scenarios suggest that the manner in which a decision is framed influences individuals to opt for genetic testing (compared with standard choice). Notably, neither satisfaction with decision nor decision regret differed across the decision frames. Perceived autonomy was an important predictor satisfaction while lack of autonomy predicted decision regret.

Genomic medicine is relevant throughout the lifespan from pre-conception (i.e., expanded carrier screening) to the newborn period (i.e., newborn bloodspot screening), to childhood/young adulthood (i.e., diagnosing Mendelian disorders), and into adult life (i.e., polygenic risk scores and cancer risk) [33]. Few studies have examined the effect of framing and genetic testing decisions. Voorwinden and colleagues examined the effect of framing and narrative information on intended participation in expanded carrier screening for autosomal recessive conditions (i.e., pre-conception carrier screening). Investigators found no significant effect on intended participation in pre-conception carrier screening [12]. Considering genetic testing in the newborn period, Lillie et al. found evidence of framing effects in the context of mandatory newborn bloodspot screening. Participants were more likely to select optional testing for a recessive condition (Duchenne muscular dystrophy) when receiving information about mandatory/standard newborn blood screening—compared with being offered testing for DMD in isolation [13]. In a proof-of-concept study using a hypothetical cancer clinical trial scenario, Abhyankar and colleagues presented participants with three frames (choice, opt-in, and opt-out) then asked participants to make a decision (i.e., enroll in the trial, pursue standard treatment, or undecided) [11]. Subsequently, participants received detailed information about the clinical trial and standard treatment and were then given the opportunity to change their initial decision. When the initial decision was presented using a default frame (opt-in or opt-out), participants were more likely to opt for the trial (or be undecided) rather than choosing standard treatment. In total, 16% of participants changed their decision after seeing detailed information. Notably, satisfaction with decision did not differ across frames—similar to the findings in the present study. Investigators concluded that presenting balanced and comprehensive information in parallel (i.e., side-by-side) prior to decision-making can help de-bias the decision frame. In contrast to Abhyankar and colleagues, participants in the present study were presented with side-by-side information prior to making a decision—yet we still observed significant framing effects. Thus, it is not clear that presenting detailed information in a side-by-side format is sufficient to de-bias decision framing. Notably, the presentation of information differed between studies. Abhyankar et al. depicted information on clinical trial vs. standard treatment more like a decision aid. Accordingly, one must be cautious not to over-interpret disparate findings between the studies.

Findings from the present study raise important questions about self-determination in genetic testing decisions. Importantly, autonomy in genetic testing may relate to the individual (i.e., agency and the right to determine what happens to an individual) as well as blood relatives [34]. For example, if an individual opts for genetic testing and gets results, the information from the test may rob blood relatives of autonomy as they may not have desired to know their potential risk. Thus, unlike other medical testing situations, genetic testing does not exist in a social vacuum—as findings also implicate blood relatives. Such ethical dilemmas are heightened by direct-to-consumer genetic testing that typically occurs without genetic counseling or clinician input [35]. Data indicate that the lay public often has high expectations regarding what genetic test results can deliver (i.e., that results are actionable) [36]. In contrast, findings of variants of unknown significance and uncertainty regarding penetrance and expressivity of variants makes interpretation challenging. Thus, genetic test results are not as definitive as the lay public perceives them to be [37]. The gap between the state of the science in interpreting genetic test results and public perception raises questions about just how informed genetic testing decisions are.

The American College of Medical Genetics has designated 73 genes as medically actionable [38]. Actionable means that finding a deleterious mutation would result in specific evidence-based medical recommendations that could reduce mortality and disease risk. Similarly, the Centers for Disease Control (CDC) advocates cascade carrier screening for “Tier 1” conditions (e.g., hereditary breast and ovarian cancer syndrome, Lynch syndrome, and familial hypercholesterolemia). Cascade carrier screening is a process for identifying, informing, and managing at-risk blood relatives of individuals at risk for heritable conditions (e.g., CDC Tier 1 conditions) [39]. By identifying potentially at-risk relatives, genetic testing can cascade through the family to inform individuals of their hereditary disease risk and guide interventions to improve outcomes. Our current findings demonstrate nudges can promote decisions for genetic testing. An ethical debate may examine the utility and appropriateness of framing decisions for Tier 1 genetic testing decisions. One may argue that framing could be applied to genetic-testing decisions because neither satisfaction nor regret are affected by choice architecture. However, as shown in Table 2, initial expectations (i.e., TPB attitudes, norms, as well as perceived consequences and behavioral control) play important roles in satisfaction and regret. This study did not assess if participants felt fully informed—another key element of high quality decisions. Thus, more clarity is needed to determine if framing encourages individuals to make less informed decisions.

The enhanced choice frame relating to norms (i.e., TPB normative beliefs) was observed to be effective for nudging individuals to opt for genetic testing. Subjective norms refer to an individual’s perception of social pressures to adopt a specific behavior [14]. Interestingly, a recent systematic review and meta-analysis examined whether subjective norms predict screening of cancer patients’ first-degree relatives [40]. Investigators found that recommendation from a physician, healthcare provider, or family/friend significantly increased the likelihood of referring for screening and/or preventive measures. Thus, it appears that normative beliefs can play an important role in decision-making as well as actions that facilitate expanding genetic screening to potentially at-risk first-degree relatives (cascade carrier screening).

It is worthwhile to note that not all genetic conditions have the same impact on health and quality of life. We compared genetic testing decision-making between two conditions with disparate prevalence (common vs. rare) and divergent disease trajectories (life-threatening vs. life-altering). Participants opting for genetic testing did not differ between the Tier 1 condition HBOC [41] and the rare-like altering condition of CHH [42]. Having prior family experience with breast cancer or a rare disease did not affect the decision for testing. Moreover, decision cognitions were strikingly similar between the hypothetical scenarios suggesting that decision-making was similar across a lethality index. Such findings hold relevance for providing decisional support for a wide range of genetic conditions regardless of prevalence or lethality. Presently, there is a shortfall of trained health professionals (i.e., genetic counselors) to meet growing genetic healthcare needs—contributing to growing health disparities [3]. To reap the full potential of genomics for improving clinical outcomes and quality of life, novel approaches are needed to extend the reach of genetic testing decisional support (i.e., telegenetics). Findings from this hypothetical experiment point to the possible role for a modular approach to decisional support in supporting high quality decisions that are informed and aligned with patient values and preferences. For example, one might imagine web-based decisional support to increase access for patients wherein set modules addressing decision cognitions could be static (i.e., eliciting and inviting reflection on “values and preferences”—how one would use the test result to inform personal/family health decisions) while disease-specific modules could be introduced to help individuals be “informed” about the disease specific to the testing situation (i.e., HBOC, CHH). Such a modular approach could be a potential scalable solution to meeting the current shortfall of genetic healthcare professionals. However, further qualitative inquiry is needed in real-life situations to determine if our findings on common/rare decision cognitions hold up beyond hypothetical testing decisions.

Findings from the present study are relevant to healthcare professionals (i.e., genetic counselors) as well as for direct-to-consumer genetic testing. First, a key tenet of genetic counseling is a non-directive approach (i.e., “choice”) that involves providing information and eliciting values and preferences to support high-quality decisions. Our observations of framing effects indicate that in hypothetical testing scenarios, testing decisions can be influenced by the way information is presented. Thus, a non-directive approach remains central for supporting patient autonomy. Moreover, our findings should caution clinicians that the way they present genetic testing information can nudge and bias patients towards testing. Similarly, study findings may be useful for informing guidelines for direct-to-consumer testing. For example, it is conceivable that marketing strategies could employ behavioral economics. Such framing of genetic testing decisions could undermine individuals’ agency and possibly be considered coercive.

A relative strength of this study is the large, relatively diverse sample that mirrors the age of individuals who are typically presented with genetic testing decisions. It merits mention that while the mean sample age reflects typical timing of HBOC testing, it is less representative of CHH (as testing often occurs between 18–20 years of age). We also utilized recommendations from the International Patient Decision Aid Standards, i.e., presenting balanced information in a side-by-side format (Supplemental Table S1), to help mitigate potential bias in presenting detailed information [43]. Study findings should be interpreted with the understanding that genetic tests are typically offered to supplement an individual’s clinical, biochemical, and/or imaging data. Thus, the decision to have genetic testing may relate to other consequences (i.e., communicating genetic risk to blood relatives, guide treatment, and informing reproductive choices) rather than whether or not an individual wishes to make/confirm a diagnosis. Limitations of this investigation include the hypothetical nature of the experiment. However, it would be unethical to manipulate the frames in a real-world setting with patients. The majority of participants were white and well educated. As such, one should be cautious in extrapolating findings to communities of color and/or populations with less than a college education. Another caveat is that the sample had a relatively high level of health literacy and numeracy. Thus, findings may not be generalizable to individuals with limited health literacy and numeracy skills.

## 5. Conclusions

In conclusion, we found framing genetic testing decisions increases the likelihood of individuals opting for genetic testing. We believe these findings have implications for non-directive genetic counseling as framing that differs from ‘choice’ may nudge individuals to have genetic testing. Findings also raise important questions about patient autonomy and self-determination in making genetic testing decisions. Examining decision cognitions revealed that perceived behavioral control is important for increasing satisfaction and minimizing regret. We neither identified differences based on disease prevalence (common/rare) nor lethality (life-altering/life-threatening), raising the possibility of a modular approach to decisional support for genetic testing.

## Figures and Tables

**Table 1:** Participant sociodemographics (*n* = 1012).

	HBOC (*n* = 507)	CHH (*n* = 505)	Total (*n* = 1012)
Age (years)			
Mean ± SD	36.1 ± 10.7	36.3 ± 10.8	36.2 ± 10.7
(95% CI)	(36.4–37.3)	(32.4–47.8)	(34.4–42.1)
Sex			
Male	304 (60%)	300 (59%)	604 (60%)
Female	203 (40%)	205 (41%)	408 (40%)
Race			
White	207 (68%)	217 (69%)	424 (68%)
Asian	73 (24%)	69 (22%)	142 (23%)
Black/African-American	19 (6%)	22 (7%)	41 (7%)
Other *	7 (2%)	8 (2%)	15 (2%)
Marital Status			
Single	242 (48%)	234 (46%)	476 (47%)
Married	265 (52%)	271 (54%)	536 (53%)
Children	246 (80%)	256 (81%)	502 (81%)
Education			
Less than college	107 (21%)	120 (24%)	227 (22%)
College graduate	306 (60%)	288 (57%)	594 (59%)
Post-graduate	94 (19%)	97 (19%)	191 (19%)
Subjective health literacy ^†^			
Adequate (n, %)	413 (81%)	409 (80%)	822 (81%)
Inadequate (n, %)	94 (19%)	96 (20%)	1990 (19%)
Objective health literacy (NVS)			
Mean ± SD	3.06 ± 0.80	2.87 ± 0.80	2.97 ± 0.06
(95% CI)	(2.90–3.22)	(2.71–3.03)	(2.85–3.08)
Past experience			
Breast cancer (n, %)	91 (18%)	n/a	91 (18%)
Rare disease (n, %)	n/a	86 (17%)	86 (17%)

HBOC: hereditary breast and ovarian cancer; CHH: congenital hypogonadotropic hypogonadism; * includes Indigenous peoples (Native American, Native Alaskan, Native Hawaiian, and Pacific Islander) and mixed race; ^†^ subjective health literacy (30,31); NVS: newest vital sign (32); n/a: not applicable.

**Table 2:** Theory of planned behavior decision cognition predictors of satisfaction with decision and decision regret.

Theory of Planned Behavior Item	Satisfaction ^†^ Bi (SE)	Regret ^‡^ Bi (SE)
Perceived risk		
This health scenario would effect me personally	B = 0.071 (0.065) *p* = 0.27	B = 0.100 (0.049) *p* = 0.042
Context/Consequences		
GT would have physical consequences for me	B = 0.018 (0.055) *p* = 0.74	B = 0.124 (0.042) *p* = 0.003
GT would have psychological consequences for me	B = 0.017 (0.053) *p* = 0.75	B = 0.049 (0.040) *p* = 0.22
GT would have social consequences for me (discrimination)	B = 0.018 (0.059) *p* = 0.76	B = 0.291 (0.045) *p* < 0.001
Attitudes		
Having GT would be an easy decision	B = 0.508 (0.069) *p* < 0.001	B = 0.258 (0.053) *p* < 0.001
Having GT would be good/bad	B = 0.195 (0.075) *p* = 0.010	B = 0.285 (0.057) *p* < 0.001
For me, having GT would be pleasant/unpleasant	B = 0.156 (0.090) *p* = 0.08	B = 0.050 (0.068) *p* = 0.46
Norms		
Having GT would be important for people I care about	B = 0.53 (0.088) *p* < 0.001	B = 0.091 (0.067) *p* = 0.17
Having GT would be important for my healthcare provider	B = 0.105 (0.059) *p* = 0.08	B = 0.083 (0.045) *p* = 0.06
For me, having GT would be valuable	B = -0.143 (0.091) *p* = 0.12	B = 0.089 (0.069) *p* = 0.20
Behavioral control		
Having GT is entirely up to me	B = 0.811 (0.085) *p* < 0.001	B = 0.126 (0.064) *p* = 0.05
If my doctor offers GT, it would be difficult for me to say no	B = -0.2 (0.045) *p* = 0.66	B = 0.031 (0.34) *p* = 0.37
I feel I have no control over my decision to have GT	B = -0.254 (0.049) *p* < 0.001	B = 0.346 (0.037) *p* < 0.001

GT, genetic testing. ^†^ Satisfaction measured by Satisfaction with Decision (SWD) score. ^‡^ Regret measured by Decisional Regret Scale (DRS) score. Multiple linear regression coefficients (B) are shown with standard error (SE).

## Data Availability

De-identified data will be made readily available upon request for research purposes to qualified individuals within the scientific community.

## Supplementary Materials

The following are available online at https://www.mdpi.com/article/10.3390/genes12060941/s1, Table S1: Information provided on disease scenarios, Table S2: Frames for genetic testing decision-making, and Table S3: Comparison of decision cognitions (TPB) between HBOC and CHH.
