# Supplementary material for: Framing Effects on Decision-Making for Diagnostic Genetic Testing: Results from a Randomized Trial
Source: Genes (Basel). 2021 Jun 20;12(6):941. doi: 10.3390/genes12060941 (PMC8234313; doi:10.3390/genes12060941)
Supplement: Supplementary file 1 [file genes-12-00941-s001.zip › genes-1248920-supplementary.pdf]

Supplemental Materials

Table S1. Information provided on disease scenarios*

Common/Life-threatening disease (hereditary breast and ovarian cancer, HBOC)

Imagine that you a have found a lump in your chest. You are worried about your health because other family members have died of cancer. You visit your doctor to discuss your concerns. The doctor takes your family history and does a physical examination. The doctor thinks the lump is cancer and tells you the following:

- The cancer is likely hereditary (caused by DNA inherited from your parents).
- This type of cancer is very serious, it is life threatening.
- You found the lump early, so there are things you can do to decrease your risk for more advanced cancer (careful monitoring and medication or surgery).
- This type of cancer runs in families. Other family members could be at risk.
- If you have children, you COULD pass cancer risk on to your children.
- Cancer is diagnosed using a blood test, imaging and a biopsy.

The doctor tells you about standard tests and genetic testing for cancer:

Standard testing	Genetic (DNA) testing
Tests<br>• A blood draw, imaging (X-rays, MRI) and a biopsy (using a needle and syringe).	*Test*<br>• Additional tube of blood for DNA testing
Cost<br>• Billed to your insurance	*Cost*<br>• Billed to your insurance
Results<br>• Results WILL determine a cancer diagnosis<br>• Results MAY change your treatment<br>• Results will NOT tell you if other family members are at risk<br>• Results will NOT tell you if you could pass cancer risk on to your children	*Results*<br>• Results are NOT needed for a cancer diagnosis<br>• Results MAY change your treatment<br>• Results MAY tell you if family members are at risk<br>• Results MAY tell you if you could pass cancer risk on to your children

Rare/Life-altering Disease (Kallmann syndrome, KS)*

Imagine that you a young adult who has not progressed normally though puberty. You look different form your peers because your puberty has been abnormal:

- If male: no beard growth, little pubic hair, voice has not deepened, no sexual drive.
- If female: no breast development, no menstrual period, no sexual drive.

You are at a visit with your doctor to discuss why your puberty is not normal. The doctor takes your family history and does a physical examination. The doctor thinks you may have a rare disease (Kallmann syndrome, KS) that is causing your abnormal puberty. The doctor tells you the following:

- KS is diagnosed by a blood test to measure your hormones.
- KS is NOT life threatening. It will NOT shorten your life.
- KS is life altering - you will take medication (hormones) for the rest of your life.
- Hormone replacement is an effective treatment for KS.
- In most cases, special treatment can give you fertility (ability to have children).
- KS is genetic (in your DNA) and can run in families. Other family members could be at risk.
- If you have children, you COULD pass KS on to your children.

The doctor tells you about hormone and genetic testing for KS.

Hormone testing	Genetic (DNA) testing
Test<br>• A blood draw to measure your hormones	*Test*<br>• Additional tube of blood for DNA testing
Cost<br>• Billed to your insurance	*Cost*<br>• Billed to your insurance
Results<br>• Results WILL determine a diagnosis<br>• Results will NOT change your treatment<br>• Results will NOT tell you if other family members are at risk<br>• Results will NOT tell you if you can pass KS on to your children	*Results*<br>• Results are NOT needed for a diagnosis<br>• Results will NOT change your treatment<br>• Results MAY tell you if family members are at risk<br>• Results MAY tell you if you could pass the condition on to your children

* NOTE: Kallmann syndrome (KS) is another term for congenital hypogonadotropic hypogonadism (CHH). For ease of participant comprehension, we used KS to describe the rare, life-altering disease scenario.

Table S2. Frames for genetic testing decision-making

Frame type	Wording
Choice (standard)	You are suitable to have a genetic (DNA) test. You have two options. Option one is to have the standard testing only. Option two is to have standard testing and a DNA test. You decide to:<br>1. have standard testing only, NO genetic (DNA) test<br>2. have standard testing AND the genetic (DNA) test
Opt-in	You are suitable to have the genetic (DNA) test. You can choose to have this test. You decide to:<br>1. have genetic (DNA) testing<br>2. NOT have genetic (DNA) testing
Opt-out	All patients automatically have a genetic (DNA) test. You are suitable for this blood test and will automatically have it done. You can decide if you do not want to have DNA testing. You decide to:<br>1. have genetic (DNA) testing<br>2. NOT have genetic (DNA) testing
Enhanced choice (context/consequences)	You are suitable to have genetic (DNA) testing for early detection. You decide to:<br>1. have genetic (DNA) testing for early detection<br>2. NOT have genetic (DNA) testing and take the risk of later diagnosis and treatment
Enhanced choice (norms)	You are suitable to have genetic (DNA) testing. Most people have a genetic (DNA) test. You decide to:<br>1. like most people, I choose to have genetic (DNA) testing<br>2. unlike most people, I choose not to have genetic (DNA) testing
Enhanced choice (affect/commitment)	You are suitable to have genetic (DNA) testing. Sharing test results with family members will inform them about their potential risk. You decide to:<br>1. have genetic (DNA) testing so I can share information with other family members who may be at risk<br>2. NOT have genetic (DNA) testing and my family members will NOT have information about their potential risk

Table S3. Comparison of decision cognitions (TPB) between HBOC and CHH

Theory of Planned Behavior item	95% CI		*p* value
	HBOC	CHH	
perceived risk			
This health scenario would effect me personally.	5.86 – 6.07	5.65 – 5.88	**0.012 ***
context/consequences			
Genetic testing would have physical consequences for me.	3.66 – 4.00	3.70 – 4.04	0.74
Genetic testing would have psychological consequences for me.	4.42 – 4.73	4.40 – 4.72	0.89
Genetic testing would have social consequences for me (e.g. discrimination).	3.36 – 3.70	3.50 – 3.83	0.27
attitudes			
Having genetic testing would be an easy decision.	5.39 – 5.63	5.48 – 5.71	0.34
Having genetic testing would be good/bad.	5.45 – 5.68	5.35 – 5.57	0.20
For me, having the genetic testing would be pleasant/unpleasant.	4.67 – 4.89	4.76 – 4.97	0.27
norms			
Having genetic testing would be important for people I care about.	5.81 – 6.03	5.61 – 5.84	**0.014 ***
Having genetic testing would be important for my healthcare provider.	5.12 – 5.37	5.02 – 5.27	0.27
For me, having genetic testing would be valuable.	5.65 – 5.88	5.56 – 5.80	0.31
behavioral control			
Having genetic testing is entirely up to me.	6.02 – 6.20	6.03 – 6.23	0.76
If my doctor offers genetic testing, it would be difficult for me to say no.	4.38 – 4.72	4.22 – 4.56	0.19
I feel I have no control over my decision to have genetic testing.	2.84 – 3.20	3.03 – 3.41	0.13

Items scored using Likert-type scale (1-7), * significant findings noted by bold and asterisk.
